# Health challenges and facilitators of arbaeen pilgrimage: a scoping review

**DOI:** 10.1186/s12889-024-17640-9

**Published:** 2024-01-09

**Authors:** Khadijeh Moulaei, Saiyad Bastaminejad, AliAkbar Haghdoost

**Affiliations:** 1 Department of Health Information Technology, Faculty of Paramedical, Ilam University of Medical Sciences, Ilam, Iran; 2 Department of Genetics, Faculty of Paramedical, Ilam University of Medical Sciences, Ilam, Iran; 3 HIV/STI Surveillance Research Center, WHO Collaborating Center for HIV Surveillance, Institute for Futures Studies in Health, Kerman University of Medical Sciences, Kerman, Iran

**Keywords:** Health, Challenges, Facilitators, Arbaeen Pilgrimage, Arbaeen walk

## Abstract

**Background:**

The Arbaeen Pilgrimage, a momentous religious journey drawing millions of participants annually, presents a profound spiritual experience. However, amidst its significance lie various health challenges that pilgrims encounter along the way. Addressing these challenges is vital to ensure the well-being of participants and the success of this extraordinary event. In light of this, the aim of this study is to examine the health challenges of the Arbaeen Pilgrimage, identify facilitators for solving these challenges, and propose effective solutions to enhance the overall pilgrimage experience for all involved.

**Methods:**

The scoping review was performed by searching databases such as Web of Science, PubMed, Scopus, and Google Scholar search engine with a focus on the keywords “Arbaeen”, “Arbaeen walk” and “Arbaeen pilgrimage”. The search was not constrained by a specific time limitation in the databases. Data from studies were extracted using a data extraction form consisting of 9 fields. The selection of articles and data extraction were carried out by two researchers, adhering to predefined inclusion and exclusion criteria. Any disagreements were resolved through consultation with a third researcher. The study was reported following the PRISMA checklist.

**Results:**

Out of 1619 retrieved articles, 9 were finally included in this study. All these studies were published since 2017 and conducted in Iraq and Iran. In total, 101 health challenges and facilitators were identified, comprising 61 challenges and 40 facilitators. The challenges with the highest frequency included “infectious disease outbreaks” (*n* = 7), “Poor management of Iraq’s health system in waste collection and disposal” (*n* = 4), “Rising incidence of walking injuries among pilgrims (e.g., burns, fractures, lacerations, wounds, and blisters)” (*n* = 4), and “Insufficient knowledge about personal and public health“(*n* = 4). The most important facilitators to solving the challenges were: “Customized pilgrim training and addressing their issues, with a focus on vital practices” (*n* = 6), “Coordinating mass gathering stakeholders, including health ministries and organizations” (*n* = 4), and “Implementing an agile syndromic system for rapid surveillance and identification of contagious illnesses” (*n* = 4).

**Conclusion:**

The article discusses health challenges faced during the Arbaeen Pilgrimage and proposes facilitative measures for participants’ well-being. It emphasizes the significance of addressing health risks in large gatherings and suggests incorporating measures for a safer and enjoyable pilgrimage experience. Overall, understanding and managing these health factors can lead to a successful execution of the Arbaeen Pilgrimage, benefiting the physical and spiritual well-being of all involved.

## Background

Arbaeen Pilgrimage is one of the largest religious gatherings in the world, commemorating the martyrdom of Imam Hussein, the grandson of Prophet Muhammad. It takes place annually in the holy city of Karbala, Iraq, attracting millions of pilgrims from different parts of the world, particularly Shia Muslims [1, 2]. The pilgrimage holds significant cultural, religious, psychological, and spiritual importance for participants who embark on a journey of mourning, reflection, and devotion [3]. During Arbaeen, pilgrims walk long distances, often on foot, to reach the holy shrine of Imam Hussein, creating a vibrant atmosphere of unity, solidarity, and reverence [4].

However, the Arbaeen Pilgrimage also presents various challenges. These challenges can cause many problems for the pilgrims and make it difficult for them to continue the journey. For instance, the sheer magnitude of participants can strain local infrastructure, leading to overcrowding and congestion along the pilgrimage routes [5]. Inadequate sanitation facilities and hygiene practices can contribute to the spread of diseases among the pilgrims. Choi et al. [5], stated that extreme weather conditions, such as scorching heat, can pose significant health risks and discomfort. Moreover, the provision of sufficient food, water, and medical assistance to the vast number of pilgrims can be a logistical challenge [6, 7]. Additional studies [8–10] have similarly highlighted those religious ceremonies like Hajj and Hinduism associated with Hindu festivals present health challenges, carrying a potential risk of transmitting infectious diseases between pilgrims and the local population. One of the ways to overcome these problems is to identify facilitators and implement measures that can help mitigate the challenges faced during the Arbaeen Pilgrimage.

According to our preliminary survey, no systematic or scoping review has been conducted to specifically identify the health challenges and incentives of Arbaeen walking. Despite the global significance of the Arbaeen Pilgrimage, a comprehensive examination of the health challenges and facilitators specific to Arbaeen walking is notably absent in the existing literature. This highlights a research gap in the current understanding of the health-related aspects of the Arbaeen Pilgrimage. Therefore, to address this gap, this scoping review was undertaken to systematically identify and analyze the health challenges and facilitators to solving these challenges inherent in the Arbaeen Pilgrimage.

## Materia and methods

### Information sources and search strategy

A search was conducted in three databases, namely PubMed, Web of Science, Scopus,and Google Scholar search engine to find articles pertaining to the health challenges of the Arbaeen Pilgrimage and facilitators for solving these challenges. The search utilized three keywords: “Arbaeen walk,” “Arbaeen pilgrimage,” and “Arbaeen.” Medical Subject Heading (MeSH) Keywords, spelling variations, and synonyms were incorporated and adjusted accordingly for each database. The search strategy was devised by KHM, and SB, and subsequently approved by AH. No specific time limitation was applied to search the databases. In order to retrieve all related articles, we used the following search strategy:

(Arbaeen walk OR Arbaeen pilgrimage OR Arbaeen)

### Eligibility criteria

#### Inclusion criteria

In this study, articles focusing on health challenges of the Arbaeen Pilgrimage and facilitators for solving these challenges, as well as articles published in English, were included.

#### Exclusion criteria

The following exclusion criteria were applied during the article selection process: (1) Articles focusing on unhealthy aspects of Arbaeen walking were excluded; (2) Articles don’t focusing on Arbaeen walking challenges and facilitators were excluded; (3) Systematic reviews, reviews, and meta-analyses were not included; (4) Books were excluded from the analysis; (5) Book chapters were not considered for inclusion; (6) Letters to the editor were excluded from the review; (7) Conference abstracts were not included in the analysis; and (8) Research protocols or protocol studies were not considered for inclusion.

#### Study selection

The retrieved studies were initially imported into Endnote X9 to facilitate the identification and elimination of duplicates. Following that, duplicates were removed from the dataset. Two researchers conducted a screening of titles and abstracts, and the full text of potentially relevant studies was independently assessed by the same two researchers. In cases where there was a discrepancy between the primary reviewers, a third researcher independently reviewed those studies, and any remaining disagreements were resolved through team discussion.

#### Data charting process and data items

The data was obtained through the utilization of a pre-established form. This form for data extraction encompasses various fields, including year, country, study type, study aim, sample size, age group, and health challenges of the Arbaeen Pilgrimage, as well as facilitators for these challenges.

### Data collation process

After obtaining final approval for the articles during the preceding steps, KHM, and SB individually read the full text of each article to extract the necessary information. The extracted information was then recorded in the aforementioned data extraction form. KHM and AH independently reviewed and verified the extracted information. In cases where discrepancies arose regarding the extracted information, the research team members convened to reach a consensus and make a final decision. It is important to note that in instances where articles lacked certain information, we reached out to the corresponding authors via email to request the missing details. Ultimately, all the extracted information was entered into an Excel spreadsheet. We implemented a systematic categorization process for the identified health challenges associated with Arbaeen walking and facilitators for solving these challenges. The challenges and facilitators were organized based on thematic similarities and underlying factors, employing a combination of deductive and inductive approaches.

### Synthesis of results

Once the data was stored and managed in MS Excel for further analysis, a thorough review was conducted by one author (KHM) to ensure the accuracy and integrity of the imported data. This involved tasks such as spell checking and formatting cells as necessary. Descriptive statistics, specifically frequency and percentage, were employed to summarize the collected data. The descriptive data derived from the findings of the included articles were then organized thematically into tables and figures. These visual representations were used to present the key findings of the review, thus serving as a guide for the study objectives, which were collectively determined by KHM, and SB.

## Results

### Selection of sources of evidence

**Fig. 1:**
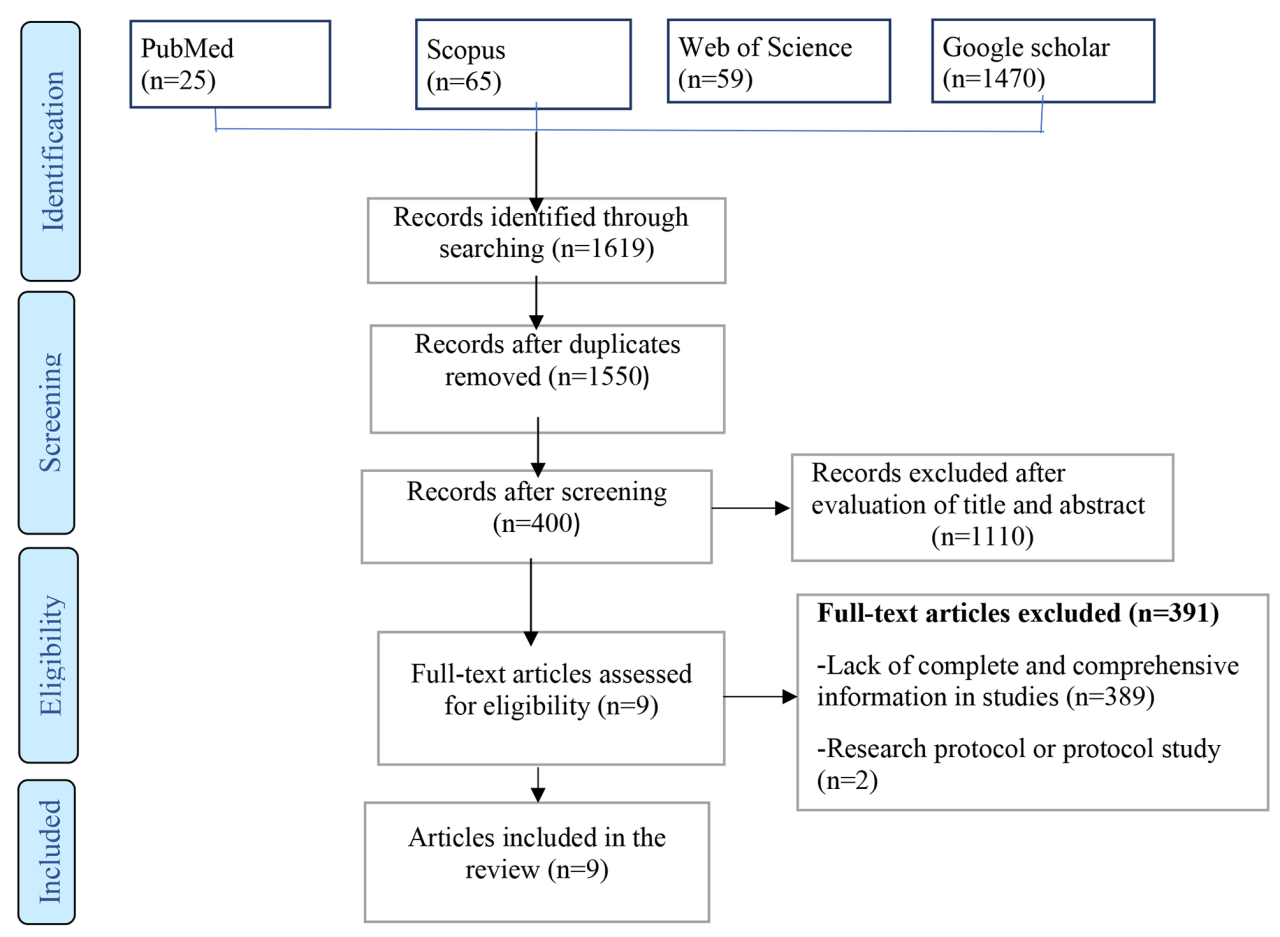
Study selection process

A total of 1619 articles were initially retrieved. Once duplicates were excluded, 1550 studies remained and underwent thorough evaluation according to specific inclusion and exclusion criteria. Ultimately, 9 articles were deemed suitable for inclusion in the study. The findings of the search and selection process can be observed in Fig. 1.

### Attributes of the included studies

Table 1 offers a comprehensive overview of the selected studies. As can be seen in this table, all of these studies have been published since 2017 and have been carried out in Iraq and Iran.

**Table 1:** A comprehensive overview of the selected studies

Ref	Year	Country	Study type	Study aim	Sample size	Age group	Male/Female
[11]	2017	Iraq	Cross-sectional	Analyzing the oral and dental well-being of individuals taking part in the Arbaeen walk.	3500	18–80	2000/1500
[7]	2018	Iran	Cross-sectional	Investigating the perspectives of stakeholders on the readiness and obstacles faced by the health system, and proposing measures to prevent infectious diseases during the Arbaeen mass gathering.	17	30–60	15/2
[12]	2019	Iraq	Cross-Sectional	Identifying the main communicable disease syndromes observed among pilgrims during the Arbaeenia mass gathering	3999		1911 / 2088
[6]	2019	Iran	Cross-sectional	Assessing the difficulties in delivering health services to pilgrims participating in the Arbaeen walk	1025	2–78	674/351
[13]	2020	Iraq	Cross-Sectional	Examining and assessing the solid waste management system implemented during the Arba’een event to bridge gaps and gain fresh insights into waste management practices during this religious gathering	9	-	-
[14]	2020	Iran	Cross-Sectional	Recognizing health challenges associated with the Arbaeen walk	18	-	-
[15]	2021	Iran	Cross-Sectional	Through exploring the perspective of the event hosts, we can ascertain the impacts of Arba’een on them and their community	22	18–55	13/9
[16]	2021	Iraq	Cross-sectional	Evaluating the effectiveness of the surveillance system and the gathered data in determining the occurrence of prevalent acute and infectious conditions, chronic illnesses, as well as trauma and injuries during the Arba’een pilgrimage.	338,399	20–49	-
[17]	2022	Iran	Mixed-method	Evaluating the possible risks linked to the Arbaeen ceremony amidst the Covid-19 pandemic in 2021	20		14/6
[18]	2022	Iran	Cross-sectional	Exploring the process of establishing a treatment camp along the Arbaeen walking route to handle patient referrals	3477	26–50	2152/1325

### Health challenges of the arbaeen pilgrimage and facilitators for solving these challenges

#### Health challenges of arbaeen pilgrimage

As Table 2 shows, a total of 61 health challenges related to Arbaeen walking were identified. These challenges were categorized into four main groups: insufficient health supervision and surveillance, inadequate health facilities and services, health risks and hazards, and lack of health knowledge and awareness. Overall, the most common challenges (based on frequency) included “Infectious disease outbreaks such as influenza“(n = 7) [6, 7, 12, 14, 16–18], “Poor management of Iraq’s health system in waste collection and disposal” (n = 4) [7, 13, 14, 17], “Rising incidence of walking injuries among pilgrims (e.g., burns, fractures, lacerations, wounds, and blisters)” (n = 4) [14, 16–18],” and “Insufficient knowledge about personal and public health.”

**Table 2:** Health challenges of arbaeen pilgrimage

Category	Sub-category (challenges)	Reference frequency	Reference
**Insufficient Health Supervision and Surveillance**	Poor management of Iraq’s health system in waste collection and disposal	4	[7, 13, 14, 17]
	Inadequate supervision of responsible organizations on health processes, for example food supply	3	[6, 7, 17]
	Poor condition of toilets and bathrooms	2	[14, 17]
	Weaknesses in Iraq’s health care system or operational and governance weaknesses	2	[6, 13]
	Infrastructural shortcomings in Iraq’s health system	2	[6, 14]
	Inadequate identification of high-risk groups	1	[17]
	Lack of precise planning Iraq’s health system	1	[6]
	Lack of health issue supervisor	1	[14]
	Mismanagement in the use of resources	1	[17]
	Weak implementation of health regulations	1	[7]
	Inadequate quarantine places for incoming patients (lack of entries’ control program)	1	[17]
	Not preparing Iraq’s health system to fulfill the health needs of the people	1	[14]
	Inability of the system to provide services in an epidemic	1	[7]
	Lack of attention to emerging and reversible illnesses	1	[7]
	Ineffectiveness of the health system in screening	1	[7]
	Incomprehensive syndromic surveillance	1	[17]
	Insufficient coverage of traffic warning signs on transportation routes	1	[17]
	Lack of attention to personal hygiene by executives and people	1	[17]
	Ignoring the gathering of people with different cultures and ethnicities	1	[7]
	Lack of sufficient equipment and distribution program	1	[17]
	Lack of comprehensive risk communication program	1	[17]
	Insufficient coverage of patient tracking system	1	[17]
	Not having a license of some healthcare provider stations	1	[14]
**Inadequate Health Facilities and Services**	Inadequate and exhausted transportation system	2	[6, 17]
	Inadequacy of workforce and facilities with needs	2	[16, 17]
	Lack of camps and resorts and overcrowding	2	[6, 17]
	Lack of access to safe water	2	[6, 14]
	Lack of trauma centers in border provinces	1	[17]
	Poor internet connection or complete lack of internet access altogether	1	[14]
	Lack of equipment to collect and dispose of waste and sewage	1	[17]
	Inadequate disinfection of resorts of pilgrims	1	[17]
	Limited availability and capacity for rapid serology tests and PCR tests, hindering extensive testing efforts to identify new cases.	1	[17]
	Scarcity of critical medical resources, including ICU beds, essential medicines, and medical equipment and supplies	1	[17]
	Drug shortages and improper distribution	1	[17]
	Deficiency of primary screening, especially at points of entry	1	[17]
	Inadequacy of qualified personnel to address healthcare needs and implement effective health processes.	1	[14]
	Inappropriate places to sleep and rest	1	[14]
	Lack of personal and public health facilities	1	[7]
	Lack of standard isolation rooms and negative pressure isolation in border hospitals and Iraq	1	[17]
	Inadequate space to settle	1	[7]
	Lack of controlled landfill site	1	[13]
**Health Risks and Hazards**	Infectious disease outbreaks such as influenza	7	[6, 7, 12, 14, 16–18]
	Rising incidence of walking injuries among pilgrims (e.g., burn, fracture, laceration, wound and blisters)	4	[14, 16–18]
	Diversity and population density	3	[6, 7, 18]
	Challenges in medication adherence among pilgrims, including refusals to take prescribed medicine, forgotten medications, running out of medications for patients with chronic diseases, and prevalence of self-treatment practices	2	[14, 17]
	Inappropriate of food and water safety and sanitary conditions	1	[14]
	Impossibility of requiring flu vaccination	1	[7]
	Heat exhaustion	1	[17]
	Improper ventilation of camps and resorts	1	[17]
	Water-borne and food-borne diseases	1	[17]
	Non-compliance with health protocols	1	[17]
	Impossibility of social distance	1	[17]
	Hot weather	1	[14]
	Air pollution	1	[7]
	Incorrect preparation, distribution, and consumption of water and food	1	[16]
	Lack of attention to health and health advice	1	[7]
	Impossibility of full coverage of vaccination of pilgrims	1	[17]
**Lack of Health Knowledge and Awareness**	Insufficient knowledge about personal and public health	4	[6, 7, 11, 17]
	Religious misconceptions about disease transmission, vaccination, etc.	1	[17]
	Lack of health belief	1	[7]
	Lack of knowledge about proper nutrition	1	[7]

### Facilitators of the arbaeen pilgrimage challenges

A total of 40 facilitators were identified in relation to solving Arbaeen challenges (Table 3). These facilitators were categorized into five main groups: comprehensive planning and coordination, health supervision and surveillance, health facilities and welfare services, health measures for pilgrims, and pilgrim training and planning. Based on frequency, the most important facilitators were: “Customized pilgrim training and addressing their issues, with a focus on vital practices like handwashing“(*n* = 6) [6, 7, 11, 13, 14, 16], “Coordinating mass gathering stakeholders, including health ministries and organizations” (*n* = 4) [6, 7, 11, 16], and “Implementing an agile syndromic system for rapid surveillance and identification of contagious illnesses” (*n* = 4) [6, 7, 12, 14].

**Table 3:** Health facilitators to solving arbaeen pilgrimage challenges

Category	Sub-category (facilitators)	Reference frequency	Reference
**Comprehensive Planning and Coordination**	Coordinating mass gathering stakeholders, including health ministries and organizations, for proper compliance with International Health Regulations (IHR) and facilitating comprehensive planning and coordination among multiple organizations before the Arbaeen pilgrimage	4	[6, 7, 11, 16]
	Effectively managing pilgrims’ financial contributions to support the construction and maintenance of health infrastructure in Iraq	1	[7]
	Implement a formal recycling scheme to improve municipal solid waste management	1	[13]
	Enhanced health support for Arbaeen pilgrimage: Iraq’s health authorities engaging in cooperative efforts with international organizations, offering equipment and facilities	1	[6]
	Developing medical guidelines for mass gatherings	1	[6]
	International aiding from the countries whose citizens attending the event are needed	1	[14]
	Provision and monitoring of food, drinks, and welfare facilities by Iraqi officials in religious gatherings for pilgrims	1	[6]
	Educate policy makers on the necessary health culture to develop a program against contagious diseases	1	[7]
	Prepare for swift patient transportation, establish on-site triage services for injured pilgrims during accidents	1	[6]
	Identify factors causing infectious disease spread among pilgrims and implement preventive measures	1	[7]
**Health Supervision and Surveillance**	Implementing an agile syndromic system for rapid surveillance and identification of contagious illnesses	4	[6, 7, 12, 14]
	Expanding syndromic surveillance to additional governorates in Iraq	2	[12, 14]
	Establishment of health monitoring systems	1	[6]
	Setting up epidemiological monitoring centers for swift laboratory diagnostic testing of specific cases like suspected measles or cholera	1	[16]
	Development of licensed care centers	1	[14]
	Development of mobile treatment and care centers	1	[14]
	Appointing a healthcare trustee to prevent unsafe food distribution and reduce infectious diseases	1	[7]
	Enabling electronic management of participants’ information	1	[6]
	Monitoring of food and drinks, as well as welfare facilities like baths, toilets, and accommodation by Iraqi officials and the health system	1	[6]
**Health Facilities and Welfare Services**	Expanding waste collection and disposal facilities by municipalities	2	[6, 14]
	Provision of necessary equipment and organization of service centers by non-governmental organizations	2	[6, 14]
	Offering travel guidelines	2	[6, 14]
	Providing access to high-speed Internet for pilgrims	1	[16]
	Free treatment for pilgrims	1	[16]
	Increasing the number of field clinics	1	[16]
**Health Measures for Pilgrims**	Vaccination to prevent infectious diseases	3	[6, 7, 16]
	Eating food from mawkibs with an indoor kitchen	1	[16]
	Considering health issues and distributing masks among pilgrims	1	[6]
	Use of hand sanitisers by participants	1	[16]
	Wearing a mask in crowded places	1	[16]
	Drinking only (sealed) packaged water	1	[16]
	Expanding the toilets and bathrooms by municipalities Developing medical guidelines for mass gatherings	1	[14]
	Distributing masks among pilgrims to prevent respiratory diseases	1	[6]
	Personal and public health practices include hand washing, sourcing food from health centers, and avoiding overeating.	1	[7]
	Encourage hygiene adherence, discourage self-treatment, and promote medication use during illness	1	[7]
**Pilgrim Training and Planning**	Customized pilgrim training and addressing their issues, with a focus on vital practices like hand washing	6	[6, 7, 11, 13, 14, 16]
	Need to inform patients and their families of the health risks	1	[14]
	Conducting specialized training courses individually for executives, and policy makers, prior to the Arbaeen event	1	[7]
	Leveraging clerics and religious figures for risk education and attitude shifts in religious gatherings	1	[6]

## Discussion

Although a significant population is both directly and indirectly affected by the Arbaeen ceremony, only a limited number of studies have delved into its effects on health. The majority of these conducted studies have focused on analyzing its potential drawbacks, revealing a consistent theme that highlights the inadequate infrastructure for delivering necessary medical services. This review is the first article that identifies health challenges and facilitators of the Arbaeen Pilgrimage. In this study, a total of 101 health challenges and facilitators were identified, with 61 challenges and 40 facilitators comprising the dataset. In the following, health challenges of the Arbaeen Pilgrimage and facilitators for solving these challenges are discussed.

### Insufficient health supervision and surveillance

Iraq’s healthcare system has faced numerous challenges that have hindered its ability to provide effective and efficient services to its citizens, particularly during critical times such as epidemics and mass gatherings like the Arbaeen pilgrimage [16]. Some studies [6, 13] have shown that weaknesses in the operational and governance aspects of the healthcare system have led to mismanagement of resources, ineffective screening, and insufficient coverage of patient tracking systems. Additionally, infrastructural shortcomings and inadequate planning have resulted in the lack of quarantine facilities and equipment distribution programs [6, 14]. Moreover, the health system has struggled to identify high-risk groups and provide comprehensive risk communication, leaving the population vulnerable to emerging and reversible illnesses.

To overcome these challenges, several facilitators can play a pivotal role in transforming Iraq’s healthcare system and enhancing its preparedness for future health crises. Coordinating with mass gathering stakeholders, including health ministries and organizations, is essential to ensure compliance with International Health Regulations (IHR) and foster comprehensive planning and coordination before events like the Arbaeen pilgrimage [6, 7, 11, 16]. Soltani et al. [6], pointed out that by involving international organizations and engaging in cooperative efforts, Iraq’s health authorities can access vital equipment and facilities to support mass gatherings effectively. Moreover, enhancing financial management of pilgrims’ contributions can channel resources into the construction and maintenance of health infrastructure, bolstering the healthcare system’s capacity [7]. Implementing formal recycling schemes can improve municipal solid waste management during crowded events, contributing to overall hygiene and cleanliness [13].

Education is crucial at multiple levels. Karampourian et al. [7], stated that policy makers need to be educated on the significance of public health and the need for comprehensive programs against contagious diseases. Additionally, promoting personal hygiene among executives and the general population can help mitigate the spread of infectious diseases [7]. Furthermore, developing medical guidelines specifically tailored for mass gatherings can help healthcare professionals in planning and responding to potential health issues during such events [14].

To address the lack of comprehensive syndromic surveillance and the inability to provide services during epidemics effectively, the health system must invest in advanced screening technologies and patient tracking systems [6, 19]. Additionally, identifying factors contributing to infectious disease spread among pilgrims and implementing preventive measures will play a significant role in protecting public health during large gatherings [7].

### Inadequate health facilities and services

Pilgrimage journeys have been an integral part of religious and cultural practices for centuries, but with them come significant challenges, particularly concerning the health and safety of pilgrims. In regions like Iraq, where the influx of pilgrims is substantial, addressing these challenges becomes even more critical. The healthcare system faces a range of obstacles, including an inadequate transportation system [6, 17], lack of essential facilities and workforce [16, 17], scarcity of resources [17], and insufficient screening capabilities [7]. However, innovative solutions can pave the way for a safer and more organized pilgrimage experience.

One of the fundamental challenges faced during pilgrimages is the inadequacy of transportation systems, leading to overcrowding and exhaustion among pilgrims [6, 17]. With millions of pilgrims converging on specific routes, the existing transportation infrastructure often struggles to cope with the overwhelming demand. To combat this, the government and relevant authorities should prioritize investing in and expanding the transportation infrastructure, ensuring smooth and efficient movement of people [7, 20]. Additionally, the scarcity of qualified healthcare personnel can be addressed by implementing training programs to build a skilled workforce capable of handling the healthcare needs of pilgrims effectively [7].

Some studies [6, 14] have shown that access to safe water and proper sanitation is a fundamental right for all individuals, especially during mass gatherings. To alleviate the lack of clean water and sewage disposal facilities, municipalities should expand waste collection and disposal facilities, while non-governmental organizations can play a crucial role in providing necessary equipment and organizing service centers to maintain hygiene standards [6, 14]. Moreover, facilitators have been working on expanding waste collection and disposal facilities, improving sanitation, and organizing service centers through non-governmental organizations, which can alleviate the burden and ensure a cleaner and more efficient environment for the pilgrims [6, 14].

Enhancing medical facilities and resources is paramount. Yousefian et al. [17], stated that the shortage of critical medical resources, drugs, and medical equipment can be mitigated by strategic planning and proper distribution. Moreover, the lack of trauma centers and isolation rooms in border provinces poses serious risks in handling emergencies and infectious diseases [17]. Setting up temporary field clinics in pilgrimage areas can help provide immediate medical attention to pilgrims and alleviate the burden on established healthcare facilities [16]. Additionally, providing free treatment for pilgrims are important facilitators in ensuring prompt medical attention and care during the pilgrimage [16].

Furthermore, the Arbaeen walk faces challenges related to communication and connectivity. Harnessing technology is vital to streamline healthcare services during pilgrimages [14]. The poor internet connection or lack thereof can hamper communication and information dissemination, making it difficult to coordinate and provide essential services [14]. However, providing access to high-speed internet for pilgrims can bridge this gap and facilitate real-time communication, emergency services, and access to necessary information [16].

### Health risks and hazards

One of the primary challenges faced during the Arbaeen walk is the potential for infectious disease outbreaks, including influenza [6, 7, 12, 16, 17]. Shafi and et al. [21], mentioned that during mass gathering religious events, there is a significant congregation of pilgrims who live and interact closely while partaking in religious rituals under crowded conditions. As a result, both the pilgrims and the local population are exposed to a variety of bacterial and viral infections. With people from different regions and backgrounds converging in close quarters, the risk of transmission increases significantly. However, facilitators have recognized the importance of vaccination as a preventive measure, encouraging pilgrims to get vaccinated against infectious diseases beforehand [6, 7, 16]. Moreover, implement a comprehensive approach comprising the establishment of health monitoring systems and the creation of epidemiological monitoring centers to enable rapid laboratory diagnostic testing for specific cases, such as suspected measles or cholera [6, 16].

Another pressing issue is the rising incidence of walking injuries, such as burns, fractures, lacerations, and blisters, among pilgrims. Long distances, intense heat, and improper footwear contribute to these injuries [16, 17]. To address this, public health initiatives have promoted personal and public health practices, including wearing comfortable shoes, using hand sanitizers, and adhering to medical guidelines for mass gatherings [14, 16–18, 22].

The Arbaeen walk’s sheer diversity and population density pose further challenges to health and safety measures, making social distancing impossible [6, 7, 17, 18]. Nevertheless, promoting hygiene practices, distributing masks, and sourcing food from reputable health centers help mitigate the risks associated with such large crowds [6].

Food and water safety, sanitation, and proper distribution are vital considerations to prevent water and food-borne diseases [14]. Encouraging pilgrims to consume food from designated mawkibs with indoor kitchens and drink only sealed packaged water can significantly reduce the risk of contamination [16].

### Lack of health knowledge and awareness

Another key challenge is the lack of knowledge about personal and public health, including proper nutrition and hygiene practices [6, 7, 11, 17]. To counter this, customized pilgrim training can play a vital role. Karampourian et al. [7], by focusing on essential practices like regular handwashing, organizers can empower participants with the knowledge needed to safeguard their health during the event. Moreover, the involvement of healthcare professionals and educators can help dispel religious misconceptions about disease transmission and vaccination, fostering a greater understanding and acceptance of preventive measures [7].

Other, challenge arises from the lack of health beliefs among some participants [7]. To tackle this, it is crucial to inform both patients and their families about the potential health risks associated with large gatherings [14]. Raising awareness about these risks can encourage individuals to take necessary precautions, ensuring a safer pilgrimage experience for all. Additionally, specialized training courses for event organizers, executives, and policymakers prior to the Arbaeen walk can enhance their capacity to manage health-related issues effectively [7]. Moreover, leveraging the influence of clerics and religious figures, who hold significant sway over participants, can be instrumental in promoting health-conscious behavior. By incorporating health-related discussions into religious discourse, misconceptions can be addressed, fostering a culture of responsibility and care among the pilgrims [6].

In the end, it should be mentioned that Arbaeen walking, as a profound religious ceremony, instills a profound sense of empathy among the pilgrims, fostering a remarkable atmosphere of mutual support and solidarity. This collective spirit generates a palpable aura of positivity and goodwill that permeates through the participants. This inherent sense of well-being and shared compassion becomes a driving force in addressing and alleviating various health challenges that may arise during the pilgrimage. Beyond its spiritual significance, this ethos encourages pilgrims to engage in acts of kindness and assistance, such as conscientiously collecting litter strewn along the route, freely distributing masks and disinfectants, providing free mineral water and refreshing drinks, offering guidance and aid to elderly pilgrims seeking medical attention, and undertaking numerous other gestures of compassion. As the pilgrimage continues to evolve, it becomes increasingly evident that the Arbaeen walk not only nourishes the soul but also serves as a testament to the potential of unity and empathy to overcome obstacles and create a harmonious community.

### Limitations of the study

There were a few limitations encountered in this review. Only studies written in English were included, and any studies published in languages other than English were not considered. To identify relevant studies, we conducted searches on three scientific databases: Scopus, PubMed, Web of Science, and the Google Scholar search engine. For more comprehensive results, future studies should expand their search to include articles published in languages other than English and encompass a broader range of databases.

## Conclusion

In conclusion, the Arbaeen walk stands as an extraordinary testament to human faith and devotion, drawing together immense crowds under unique circumstances that exert notable influences on individual well-being. While comprehensive research on its health implications remains scarce, existing investigations have shed light on the anticipated health challenges posed by this massive congregation. Addressing the identified challenges is crucial to ensure the safety and well-being of the millions of participants. The challenges and facilitators identified in this study shed light on the complexities of managing health during the Arbaeen pilgrimage in Iraq. Addressing the identified challenges and implementing the facilitators can significantly improve the health system’s capacity to safeguard the well-being of pilgrims and the local population during this significant religious event. Collaborative efforts among stakeholders, comprehensive planning, and proactive measures are essential to enhancing the health response and ensuring a safe and healthy pilgrimage experience.

Yet, overshadowed by these concerns are the unexplored and potentially profound positive effects that this religious observance might have on the holistic health of pilgrims. The interplay between physical exertion, mental rejuvenation, social cohesion, and above all, the enrichment of spiritual health within the context of this spiritual journey opens a compelling avenue for future inquiry, inviting a deeper understanding of the Arbaeen walk’s intricate impact on the human condition.

## Data Availability

All data generated or analyzed during this study are included in this published article.

## Abbreviations

MeSH: Medical Subject Heading
